# Évaluation du GeneXpert^®^ MTB/RIF dans le diagnostic moléculaire de la tuberculose et de la résistance à la rifampicine en Algérie

**DOI:** 10.48327/mtsi.v4i2.2024.301

**Published:** 2024-06-12

**Authors:** Ferroudja YAMOUNI, Fatma Zohra HENNICHE, Malika IFTICENE, Madjid CHABANI, Doria BENSERSA, Nour el Houda OUADAH, Mansuer NIHAD, Ali ZEROUKI

**Affiliations:** 1 Laboratoire de microbiologie, Hôpital central de l’armée « Mohamed Seghir Nekkache », Alger, Algérie; 2 Laboratoire national de référence de la tuberculose et de la surveillance de la résistance, Institut Pasteur d’Alger, Algérie

**Keywords:** Tuberculose, GeneXpert^®^ MTB/RIF, Résistance à la rifampicine, Complexe *Mycobacterium tuberculosis*, PCR, Algérie, Maghreb, Tuberculosis, GeneXpert^®^ MTB/RIF, Resistance to rifampicin, *Mycobacterium tuberculosis* complex, PCR, Algeria, Maghreb

## Abstract

**Objectif:**

ITB/RIF (GX) dans le diagnostic de la tuberculose pulmonaire et extra-pulmonaire par rapport à la culture bactérienne, et comparer les résultats de détection de la résistance à la rifampicine du GX par rapport au test de sensibilité phénotypique.

**Matériel et méthodes:**

Étude rétrospective d’évaluation du test GX, réalisée sur une période de cinq ans allant de mai 2017 à juin 2022, par le laboratoire de microbiologie de l’Hôpital central de l’armée Mohamed Seghir Nekkache à Alger. Les échantillons cliniques pulmonaires et extra-pulmonaires ont été recueillis, mis en culture, et ont fait l’objet d’une PCR GX. L’examen direct a été effectué par coloration de Ziehl-Neelsen. L’étude de sensibilité aux antituberculeux a été réalisée selon la méthode des proportions sur milieu liquide « Bactec MGIT 960 » (ou sur milieu solide Lowenstein-Jensen à l’Institut Pasteur d’Algérie).

**Résultats:**

310 prélèvements ont été inclus dans l’analyse finale de l’étude. Au total, le complexe *Mycobacterium tuberculosis* (MTBC) a été détecté dans 95 prélèvements (dont 84 étaient positifs par le GX) provenant de 88 patients tuberculeux. La sensibilité, la spécificité, la valeur prédictive positive et la valeur prédictive négative du GX par rapport à la culture pour les échantillons pulmonaires et extra-pulmonaires étaient respectivement de 96,3 % *vs* 77 %, 98 % vs 99,1 %, 96,2 % vs 96,5 % et 98 % vs 92,7 %. La sensibilité du GX pour la tuberculose discovertébrale, ganglionnaire, méningée et pleurale était respectivement de 100 %, 90 %, 71,4 % et 57,1 %. La sensibilité du GX pour la tuberculose pulmonaire comparée à la microscopie était de 96 % *vs* 68 %. La comparaison des résultats de détection de la résistance à la rifampicine par le GX et par méthodes phénotypiques a montré une concordance parfaite.

**Conclusion:**

Dans la présente étude, une très bonne sensibilité de GX comparé à la microscopie a été révélée. Ce test a été d’un grand apport dans le diagnostic de la tuberculose pulmonaire particulièrement à frottis négatif. La sensibilité du GX dans le diagnostic de la tuberculose extra-pulmonaire est variable selon la localisation de la tuberculose. Un résultat négatif par le GX n’exclut pas la tuberculose. De plus, les cas de résistance à la rifampicine détectés par le GX doivent être confirmés par méthode phénotypique.

## Abstract (en)

**Objective:**

1) To evaluate the contribution of the GeneXpert^®^ MTB/RIF (GX) test in the diagnosis of pulmonary and extra-pulmonary tuberculosis compared to culture. 2) To compare the rifampicin results resistance obtained by GX with the phenotypic sensitivity test.

**Materials and methods:**

Retrospective study carried out over a period of five years, from May 2017 to June 2022 at the microbiology laboratory of the Central army Hospital Mohamed Seghir Nekkache, Algiers (Algeria). The pulmonary and extrapulmonary clinical specimens were collected, cultivated, tested by GX PCR and direct examination by Ziehl-Neelsen staining. The study of sensitivity to antituberculosis drugs was performed according to the proportion method on liquid medium Bactec MGIT 960 (or on solid medium Lowenstein-Jensen at the Algerian Pasteur Institute).

**Results:**

310 samples were included in the final analysis of the study, of which 156 were of pulmonary origin and 154 of extrapulmonary origin. *Mycobacterium tuberculosis* complex (MTBC) was detected in 95 samples from 88 tuberculosis patients (sex ratio 2,03 and middle age 37 years) with 49 cases of pulmonary tuberculosis and 39 cases of extra-pulmonary tuberculosis. For 2 cases, the GX was positive while the culture was negative and for 11 cases, the GX was negative while the culture was positive. Thus, in our study and compared to culture, GX showed an overall sensitivity of 88.2%, a specificity of 98.6%, a positive predictive value (PPV) of 96.4% and a negative predictive value (NPV) of 95.2%. The analysis of the data according to the type of samples, the sensitivity, specificity, PPV and NPV of GX for the pulmonary and extrapulmonary samples were 96.3% vs. 77.0%, 98.0% vs. 99.1%, 96.2% vs. 96.5% and 98.0% vs. 92.7% respectively. The sensitivity of GX for disco-vertebral, lymph node, meningeal and pleural tuberculosis were 100%, 90.0%, 71.4% and 57.1% respectively. The sensitivity of GX for pulmonary tuberculosis compared to microscopy was 96% vs. 68%. The comparison of the results of detection of resistance to rifampicin by GX and by phenotypic methods showed perfect agreement.

**Discussion and conclusion:**

A good sensitivity of GX compared to microscopy was revealed. The GX is a useful tool for the diagnosis of pulmonary tuberculosis, especially in smear-negative cases. The sensitivity of GX in extrapulmonary tuberculosis varied depending on the location of the infection. A negative result by GX does not exclude tuberculosis and cases of resistance to RIF detected by GX must be confirmed by phenotypic method.

## Introduction

*Mycobacterium tuberculosis* représente l’agent principal de la tuberculose humaine, qui constitue un véritable problème de santé publique [2]. À l’échelle mondiale, la tuberculose est la 13^e^ cause de mortalité et la deuxième due à une maladie infectieuse, derrière la Covid-19 (et avant le sida). En 2020, selon l’Organisation mondiale de la santé (OMS), 9,9 millions de personnes auraient contracté la maladie avec 1,5 million de décès dont 214 000 coinfectées par le VIH [22]. Pour la tuberculose pharmaco-résistante, l’OMS a estimé en 2019 un taux de résistance à la rifampicine (RIF) de 5,1 % (TB-RR) dont 78 % pour la tuberculose multirésistante (TB-MR) [21]. L’Algérie reste un pays endémique. En 2021, 18 825 nouveaux cas de tuberculose ont été déclarés avec 29 % d’origine pulmonaire et 71 % d’origine extrapulmonaire, soit une incidence de 42,4 pour 100 000 habitants [23]. A l’échelle nationale, le nombre de cas de tuberculose résistante aux anti-bacillaires a fortement diminué de 1967 à 2011 grâce au plan national de lutte contre la tuberculose [1, 15]. Les techniques conventionnelles de diagnostic de la tuberculose présentent chacune un certain nombre de limites : faible sensibilité de la microscopie, risque de contamination croisée et long délai d’obtention des résultats de la culture [8, 9]. Face à ces difficultés, en décembre 2010, l’OMS a approuvé l’utilisation du test GeneXpert^®^ MTB/RIF (GX) qui a permis non seulement un diagnostic rapide, mais qui a aussi amélioré la sensibilité diagnostique par rapport à la microscopie, grâce à la recherche, à partir d’échantillons cliniques, de faibles quantités de génome du complexe *M. tuberculosis* (MTBC) et des mutations qui déterminent la résistance à la rifampicine [12, 13, 14].

Notre étude vise principalement à évaluer les performances diagnostiques du GeneX-pert^®^ MTB/RIF (Cepheid Sunnyvale, CA, États-Unis) dans le diagnostic rapide de la tuberculose pulmonaire « TP » et extra-pulmonaire « TEP » par rapport à la culture *(gold standard)* [19]. Elle vise, secondairement, à comparer les résultats de la détection de la résistance à la rifampicine du GX par rapport au test de sensibilité phénotypique.

## Matériel et méthodes

### Type - Période - Cadre de l’étude

Il s’agit d’une étude rétrospective d’évaluation du test GeneXpert^®^ MTB/RIF par rapport à la culture bactérienne, réalisée sur une période de cinq ans allant de mai 2017 à juin 2022 au niveau de l’Hôpital central de l’armée Mohamed Seghir Nekkache d’Alger. Les tests PCR GX ont été effectués sur demande explicite des cliniciens qui ont accompagné les prélèvements d’une fiche de renseignements comportant notamment les signes cliniques et radiologiques, la notion de contage et le résultat des IDR à la tuberculine ou ceux des Quantiferon^®^.Tous les prélèvements reçus au laboratoire pendant la période de l’étude n’ont pas été retenus, seuls les échantillons répondant aux critères de sélection suivants ont été inclus : échantillons provenant de patients présentant des signes de tuberculose pulmonaire ou extra-pulmonaire évolutive dont le diagnostic était fortement suspecté devant la conjonction d’arguments cliniques et radiologiques; échantillons provenant de patients présentant une suspicion d’échec thérapeutique ou d’une rechute; échantillons venant de patients posant un problème de diagnostic.

### Traitement des échantillons et techniques de détection du complexe *tuberculosis*

Les échantillons cliniques ont bénéficié d’un test GX, d’un examen direct et d’une culture. Chaque échantillon est accompagné d’une fiche de renseignements.

Pour l’examen microscopique, les frottis sont réalisés directement à partir du prélèvement, et après fixation à la chaleur, une coloration de Ziehl-Neelsen est réalisée par l’automate RAL Stainer^®^. Sous microscopie optique au grossissement 100, les BAAR apparaissent en rose sur un fond bleu.

Pour la culture, un prétraitement a été adopté selon le type d’échantillon. Les échantillons non stériles ont été décontaminés par la soude (NaOH) à 4 % selon la méthode de Petroff (crachat, tubage gastrique, liquide d’aspiration bronchique, liquide broncho-alvéolaire, urine, pus fistulisé et biopsie). En revanche, les échantillons stériles (liquide céphalorachidien, liquide articulaire, liquide péricardique, liquide pleural, liquide d’ascite, liquide péritonéal) ont été ensemencés directement dans le milieu de culture. Un ensemencement d’un volume de 0,2 ml sur milieu solide Lowenstein-Jensen (LJ) et de 0,5 ml sur le milieu liquide MGIT (système MGIT Bactec 960, BD, USA) a été effectué. Un aspect typique des colonies de *M. tuberculosis* rugueuses, à bords irréguliers en chou-fleur a été obtenu sur milieu LJ. Les résultats positifs sur MGIT ont été confirmés par le test immunochromatographique TB Ag MPT64 qui détecte l’antigène MPT64 spécifique du complexe *M. tuberculosis* [9].

### Test de sensibilité aux antituberculeux

Des tests de sensibilité aux anti-bacillaires ont été effectués par la méthode des proportions, en mesurant la croissance de MTBC en présence d’antituberculeux sur milieu liquide Bactec MGIT 960 (ou sur milieu solide Lowenstein Jensen à l’Institut Pasteur d’Algérie).

### Test GeneXpert^®^ MTB/RIF

#### Principe du test

Le GeneXpert^®^ MTB/RIF (GX) est un test diagnostique, automatisé, par PCR nichée en temps réel, conçu pour la détection simultanée du complexe MTB et de la résistance à la rifampicine, avec une estimation semi-quantitative de la concentration du génome (élevée, moyenne, faible et très faible) en moins de deux heures, à partir d’échantillons cliniques [4, 14] La région centrale cible de 81 Pb du gène *rpoB* est amplifiée et couplée avec cinq balises moléculaires ou probes (A, B, C, D et E) [14]. Chaque sonde est complémentaire d’une séquence cible différente dans le gène *rpoB* de MTBC sensible à la rifampicine (souche sauvage) et est marquée avec un fluorophore de couleur différente [14]. Une mutation au sein de ces séquences interfère avec l’hybridation de sorte que l’intégrité conformationnelle de la sonde peut être conservée à l’état non fluorescent [14].

#### Procédure et préparation des échantillons

Les échantillons cliniques sont traités avec un réactif d’échantillon *Sample Reagent (SR),* composé du NaOH et de l’isopropanol [4]. Le SR est ajouté dans un rapport de 2:1 à l’échantillon, homogénéisés au vortex puis incubé pendant 15 minutes à température ambiante. Cette étape est conçue pour liquéfier les échantillons et réduire la viabilité de MTBC afin de réduire le risque biologique [11]. Un volume de 2 ml de l’échantillon traité est transféré à l’aide d’une pipette jetable stérile dans la chambre de la cartouche qui est ensuite chargée dans l’instrument GeneXpert. Le traitement ultérieur est entièrement automatisé.

#### Analyses statistiques

Les données des patients liées au sexe et à l’âge ainsi que les résultats du diagnostic de la tuberculose par les méthodes utilisées ont été saisies et analysées avec le tableur Microsoft^®^ Excel^®^ 2013.

La sensibilité, la spécificité, la valeur prédictive positive et la valeur prédictive négative du GeneXpert^®^ MTB/RIF ont été calculées par rapport à la culture bactérienne.

## Résultats

### Identification du complexe *Mycobacterium tuberculosis* (MTBC)

**Figure 1:**
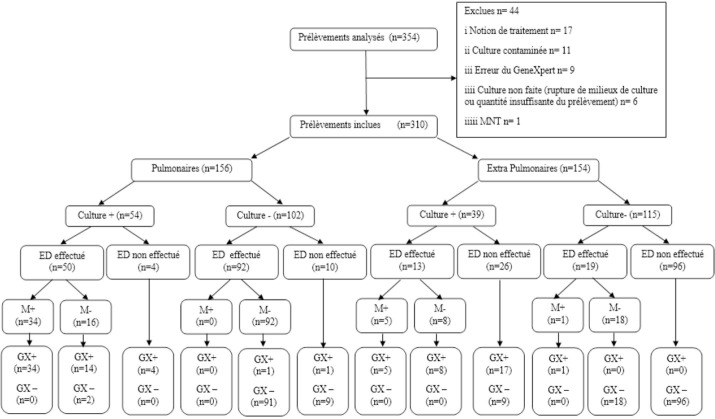
Caractéristiques des résultats des échantillons de l’étude suivant les techniques utilisées Characteristics of the results of the samples of the study according to the techniques used

**Figure 2:**
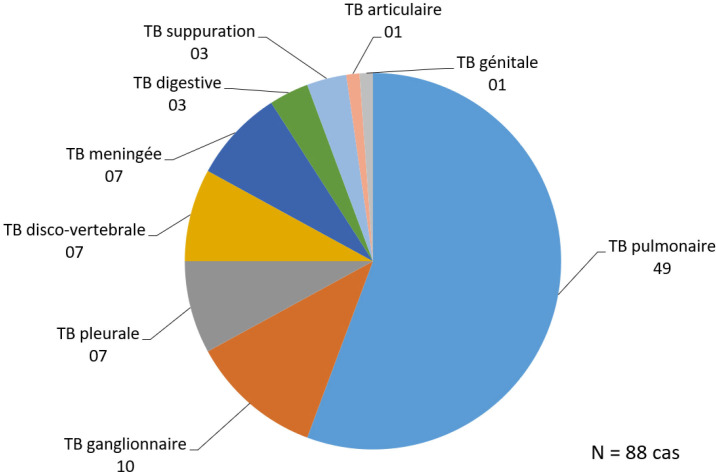
Répartition du nombre de cas de tuberculose pulmonaire et extra-pulmonaire Distribution of the number of cases of pulmonary and extrapulmonary tuberculosis

Sur un nombre total de 354 échantillons analysés (173 d’origine pulmonaire et 181 d’origine extra-pulmonaire), 44 ont été exclus pour des raisons diverses, et 310 prélèvements ont été inclus dans l’analyse finale de l’étude dont 156 d’origine pulmonaire et 154 d’origine extra-pulmonaire. Les caractéristiques des résultats des échantillons de l’étude suivant les techniques utilisées sont détaillées Figure 1. Parmi les 310 prélèvements analysés, le MTBC a été détecté dans 95 prélèvements provenant de 88 patients. La répartition du nombre de cas de tuberculose pulmonaire et extra-pulmonaire est représentée Figure 2. L’âge moyen des patients était de 37 ans avec un intervalle de confiance à 95% [33,08 - 40,90]. Le sex-ratio était de 2,03 (59 hommes contre 29 femmes). Les taux de positivité pour la culture, la mi croscopie et le GeneXpert^®^ MTB/RIF étaient respectivement de 30 % (93/310), 23 % (40/174) et 27,4 % (85/310). Ces taux étaient respectivement de 34,6 % (54/156), 24 % (34/142) et 34,6 % (54/156) pour les prélèvements pulmonaires et de 25,3 % (39/154), 18,8 % (6/32) et 20,1 % (31/154) pour les prélèvements extrapulmonaires. Le taux de positivité des méthodes de diagnostic est représenté Tableau I.

**Tableau I:** Taux de positivité des méthodes de diagnostic selon la localisation de la tuberculose Positive rate of diagnostic methods according to the location of tuberculosis

Prélèvements/méthodes	Culture LJ/MGIT %	Microscopie ZN %	GeneXpert^®^MTB/RIF %
Prélèvements pulmonaires	34,6	24,0	34,6
Prélèvements extra-pulmonaires	25,3	18,8	20,1
Total	30	23	27,4

### Performances du test GeneXpert^®^ MTB/RIF

Par rapport aux résultats de la culture, la sensibilité, la spécificité, la valeur prédictive positive (VPP) et la valeur prédictive négative (VPN) globales du GX étaient de 88,2 %, 98,6 %, 96,5 %, 95,1 %. Pour les échantillons pulmonaires et extra pulmonaires, les sensibilités du test GX étaient respectivement de 96,3 *%* et 77 *%* (Tableau II). Les performances du GX selon les résultats de la culture en fonction du type de prélèvements sont résumées Tableau III.

**Tableau II:** Sensibilité, spécificité, valeur prédictive positive et valeur prédictive négative du GeneXpert^®^ par rapport à la culture selon la localisation de la tuberculose Sensitivity, specificity, positive predictive value, and negative predictive value of the GeneXpert^®^ according to the location of tuberculosis

Localisation	Sensibilité %	Spécificité%	VPP %	VPN %
Pulmonaires	96,3	98,0	96,2	98,0
Extra-pulmonaires	77,0	99,1	96,5	92,7

**Tableau III:** Performances du GeneXpert^®^ en fonction du type de prélèvements Performance of the GeneXpert^®^ according to the type of samples

Types de prélèvements	n	GeneXpert	
		Sensibilité %	Spécificité %
Crachat	132	100 (n=47/47)	98,7 (n=75/76)
Tubage gastrique	16	100 (n=5/5)	100 (n=11/11)
Liquide d’aspiration bronchique	15	0 (n=0 /2)	100 (n=13/13)
Liquide broncho alvéolaire	1	/	/ (n=1/1)
Total des prélèvements pulmonaires	155	96,30 (n=52/54)	99 (n=100/101)
LCR	32	71,4 (n=5/7)	100 (n=25/25)
Liquide pleural	32	57,1 (n=4/7)	100 (n=25/25)
Liquide péritonéal	24	33,3 (n=1/3)	100 (n=21/21)
Pus	22	100 (n=3/3)	100 (n=19/19)
Pus ganglionnaire	15	88,9 (n=8/9)	100 (n=6/6)
Urine	11	0 (n=0/1)	100 (n=10/10)
Pus disco-vertébral	7	100 (n=7/7)	/
Liquide articulaire	5	100 (n=2/2)	100 (n=3/3)
Biopsie	3	/	100 (n=3/3)
Total des prélèvements extra-pulmonaires	153	76,92	100

La sensibilité du GX comparée à celle de la microscopie pour les cas de tuberculose pulmonaire était respectivement de 96 % (48/50) vs 68 % (34/50). Les sensibilités du GX et de la microscopie (la culture bactérienne étant le *gold standard)* pour les prélèvements pulmonaires sont résumées dans le tableau IV.

**Tableau IV:** Sensibilité du GeneXpert^®^ et la microscopie pour les prélèvements pulmonaires The sensitivity of GeneXpert^®^ and microscopy of lung samples

	Sensibilité %
Microscopie	68
GeneXpert	96
M+	100
M-	87,5

### Détection de la résistance à la rifampicine

Les résultats du diagnostic de résistance à la rifampicine par GeneXpert^®^ MTB/RIF pour 95 prélèvements ont montré 86 cas de tuberculose sensible, contre 6 cas résistants de tuberculose pulmonaire et 3 résultats indéterminés. La comparaison des résultats du GX pour un total de 24 échantillons par rapport aux méthodes phénotypiques, a montré une parfaite concordance pour 22 échantillons (16 cas sensibles et 6 cas résistants) et 2 résultats indéterminés par le GX, détectés sensibles par l’antibiogramme.

Pour les six cas résistants à la RIF détectés par le GX, les profils de résistance obtenus par méthode phénotypique étaient les suivants : trois cas de tuberculose multi-résistante (MDR) (résistance à l’isoniazide et à la rifampicine), deux cas de tuberculose pré-ultra résistante (pré-XDR) (résistance à l’ofloxacine en plus de la multi résistance) et un cas de tuberculose ultra-résistante (XDR) (résistance à l’ofloxacine et la kanamycine en plus de la multi résistance).

### Temps de détection (délai moyen d’exécution)

Le délai moyen de détection du MTBC et de la résistance à la rifampicine était de deux heures pour le GeneXpert contre 28 - 42 jours pour la culture sur LJ (10 à 28 jours sur MGIT) avec un mois supplémentaire pour détecter la résistance à la RIF par méthode phénotypique sur milieu solide (12 jours sur MGIT).

## Discussion

En matière de soins de la tuberculose et de lutte antituberculeuse, les priorités mondiales sont d’améliorer la détection des cas de manière précoce et de renforcer la capacité à diagnostiquer la tuberculose résistante [20]. Dans la présente étude, la sensibilité et la spécificité du GX dans la détection de la tuberculose pulmonaire et extra pulmonaire ainsi que la résistance à la rifampicine ont été évaluées. Les résultats de notre étude ont montré que la pratique d’un test GX a un apport considérable dans la détection du MTBC, essentiellement dans les prélèvements respiratoires et dans la détection de la résistance à la rifampicine. En considérant la culture comme *gold standard,* nous avons constaté une différence de sensibilité du GX selon la localisation de la tuberculose. Une bonne sensibilité pour la localisation pulmonaire a été révélée. Des résultats similaires ont été rapportés par la plupart des études dans la littérature [13, 26]. Elbrolosy *et al.* en Egypte ont trouvé une sensibilité de 90,2 % pour la localisation pulmonaire et 81,6 % pour la localisation extrapulmonaire [6].

Le diagnostic de la tuberculose extra-pulmonaire (TEP) reste un défi pour les cliniciens et les microbiologistes en raison de sa variabilité clinique et du caractère paucibacillaire des prélèvements [17]. La proportion élevée des prélèvements d’origine extra-pulmonaire dans notre étude, s’explique par l’évolution de l’épidémiologie de la tuberculose en Algérie ces dernières années : actuellement, 70 % des cas enregistrés de tuberculoses sont de localisation extra-pulmonaire contre 30 % de localisation pulmonaire.

Le taux de sensibilité du GX varie selon le site de la TEP. Dans notre étude, on a trouvé une bonne sensibilité pour la tuberculose ganglionnaire, disco-vertébrale et les suppurations mais une sensibilité modérée pour la tuberculose pleurale et méningée. Cependant, la sensibilité était faible pour la tuberculose péritonéale. Cette variation de la sensibilité du test GX selon le type de prélèvement s’expliquerait par le fait que la charge mycobactérienne qui est variable selon les différents compartiments du corps serait le principal déterminant de la positivité du test GX [5]. Dans les autres études, la sensibilité du GX est aussi variable en fonction de la localisation de la TEP. Cependant, il est très difficile de comparer les résultats, en raison de l’hétérogénéité de la population étudiée, des critères de sélection des patients, du type de TB, de la qualité et la nature des échantillons et du *gold standard* utilisé dans les études [17]. Dans les différentes études, le GX donne des résultats corrects pour les suppurations et les biopsies notamment ganglionnaires et osseuses. En revanche, les résultats sont très controversés pour les liquides d’épanchement et l’urine [8,13,16,17,18,25]. Dans la présente étude, le GX a montré une sensibilité supérieure à celle de la microscopie dans le diagnostic de la tuberculose pulmonaire. Nos résultats se rapprochent de ceux obtenus par Hassan *et al.* [10] et Theron *et al.* [24] qui ont retrouvé respectivement une sensibilité de 77,3 % et 55 % parmi les échantillons testés M (-). Cette différence de sensibilité entre le GX et la microscopie s’expliquerait par le fait que la limite de détection du GX est de 131 CFU/ml tandis que celle de la microscopie est comprise entre 5 000 et 10 000 CFU/ml [5]. Pour les prélèvements pulmonaires, le GX a permis de récupérer 88,2 % (15/17) des prélèvements négatifs à la microscopie, d’où son intérêt pour le diagnostic de la tuberculose pulmonaire à frottis négatif. La sensibilité du GX de la présente étude et celles des différentes études de la littérature sont résumées Tableau V.

**Tableau V:** Sensibilité du GeneXpert^®^ de la présente étude et celles des différentes études de la littérature Sensitivity of the GeneXpert^®^ of the present study and in various studies

Études	Notre étude Algérie	Elbrolosy [6] Égypte	Hassan [10] Maroc	Mechal [17] Maroc	Kohli [13]	Khan [12] Pakistan	Ghariani [8] Tunisie
Nombre de prélèvements	310	582	132	714	Meta analyse	737	174
Année	2017/2022	2021	2021	2019	2018	2018	2015
Prélèvements pulmonaires	96,3 %	90,2 %	56,6 %	79,3 %	/	/	/
M+	100 %	/	100 %	/	/	/	
M-	87,5 %	/	77,3 %	/	/	/	
Prélèvements extrapulmonaires	77 %	81,6 %	/	78,2 %	/	/	/
Pus disco-vertébral	100 %	/	/	/	> 80 %	/	/
Pus ganglionnaire	90 %	/	/	87,5 %	/		87,5 %
LCR	71,4 %	/	/	85,7 %	60,9–80,4 %	83 %	/
Liquide pleural	57,1 %	/	/	/	60,9–80,4 %	58 %	/

Parmi les 95 prélèvements (MTBC+) provenant de 88 patients tuberculeux, la culture a détecté 93 prélèvements positifs contre 84 pour le GX. Le GX a permis de récupérer 2 cas positifs (1 TP et 1 TB ganglionnaire) diagnostiqués par un faisceau d’arguments, détectés négatifs par la culture. La négativité de la culture peut être expliquée par : (i) la prise éventuelle d’antibiotiques tels que les fluoroquinolones ou les anti-bacillaires avant le prélèvement; (ii) la décontamination agressive par la soude NaOH 4 % qui est susceptible de détruire les bacilles tuberculeux présents dans l’échantillon; et (iii) la nature paucibacillaire des prélèvements extra-pulmonaires avec une tendance de *M. tuberculosis* à former des amas, ce qui conduit à une répartition inégale des bacilles [9, 16].

La négativité de la PCR pour 11 prélèvements (2 TP, 9 TEP, dont 3 cas de TB pleurale, 2 cas de TB méningée, 2 cas de TB digestive, 1 cas de TB dico-vertébrale et 1 cas de TB génitale) détectés positifs par la culture s’expliquerait par : (i) la présence des inhibiteurs de la réaction de PCR dont le sang, le pus (globules blancs), l’ADN humain, le tabac et certains médicaments tel que les corticostéroïdes [14]; (ii) la limite de détection de la culture qui est de 10–100 CFU/ml, inférieure à celle du GX qui est de 131 CFU/ml [5]. Un résultat faussement positif sur un prélèvement de crachat a été détecté par le GX avec un taux très faible pour lequel le diagnostic de la tuberculose a été exclu. Durant notre étude, parmi les 44 prélèvements exclus, nous avons isolé une mycobactérie atypique à partir d’un crachat, non détectée par le GX, alors que l’examen direct était positif.

La détection rapide de la résistance à la rifampicine (RIF) permet la mise en place immédiate d’un traitement adapté et améliore le pronostic du malade tout en réduisant les risques de transmission des souches résistantes.

Dans la présente étude, la comparaison des résultats de détection de la résistance à la RIF obtenus par le GX et les méthodes phénotypiques a montré une parfaite concordance. Cependant, de rares résultats faussement positifs pour la résistance à la rifampicine ont été rapportés par des études antérieures [5, 16]. De ce fait, il est recommandé de confirmer les cas de résistance par des tests de sensibilité aux antituberculeux avant l’initiation du traitement [3].

Notre étude présente une série de limites : (i) le faible nombre d’examens directs effectués (32/154) pour les échantillons extra-pulmonaires nous a empêchés de tirer des conclusions sur les performances du GX par rapport à la microscopie pour la TEP; (ii) le manque d’évaluation de la précision diagnostique du test GX sur des échantillons autres que ceux testés dans cette étude ou testés en nombre faible, lié au coût élevé du test limitant son utilisation en routine; et (iii) étant donné que les tests de sensibilité par méthode des proportions ont été effectués pour 24 souches seulement, nous n’avons pas été en mesure d’évaluer les performances du GX dans la détection de la résistance à la rifampicine.

## Conclusion

Notre étude consistait à évaluer les performances diagnostiques du test moléculaire GeneXpert^®^ MTB/RIF dans le diagnostic précoce de la tuberculose pulmonaire et extra pulmonaire, ainsi que dans la détection rapide de la résistance à la rifampicine. Nos résultats font ressortir que le GX est d’un apport considérable dans le diagnostic de la tuberculose et la détection de la résistance à la rifampicine, problème de plus en plus préoccupant. Ce test entraine un raccourcissement spectaculaire du temps de détection permettant la prise en charge médicale précoce des patients conformément aux recommandations de l’OMS. Cette étude a révélé une très bonne sensibilité du GX comparé à la microscopie. Ce test a été d’un grand apport dans le diagnostic de la TP particulièrement à frottis négatif. La sensibilité du GX dans le diagnostic de la TEP a été variable selon la localisation de la tuberculose. Le GeneXpert présente l’avantage d’être facile à utiliser, moins dépendant des compétences de l’utilisateur, et de réduire considérablement tout risque de contamination grâce à son système fermé. Cependant, malgré ses avantages, le GX possède l’inconvénient d’être coûteux. Le coût d’un examen par GeneXpert est de 3 493 dinars algériens (DA) et celui de la culture est de 400 DA.

De plus, un résultat négatif n’exclut pas le diagnostic de la tuberculose, et la détection de la résistance à la rifampicine doit être confirmée par méthode phénotypique. Néanmoins, il n’existe actuellement aucun test de référence parfait. L’utilisation systématique du GX couplée à la culture et associée aux données cliniques, radiologiques et histologiques permettrait de mieux diagnostiquer la tuberculose dans notre pays où la tuberculose est endémique. Le test GX mériterait d’être utilisé comme test diagnostique initial en cas de suspicion de TB pulmonaire ou de TB multi-résistante et nos résultats encouragent son intégration dans le programme national de lutte contre la tuberculose.

## Contribution des auteurs

YAMOUNI Ferroudja : conception et conduite de l’étude, supervision des travaux du début à la fin et rédaction du manuscrit.

MANSEUR Nihed et OUADAH Nour El Houda : collecte des données, analyses de laboratoire et analyses statistiques.

IFTICENE Malika : réalisation et lecture des antibiogrammes.

HENNICHE Fatma Zohra, CHABANI Abdelmadjid, BENSERSA Doria et ZEROUKI Ali : analyse critique du manuscrit.

Tous les auteurs ont lu et approuvé la version finale du manuscrit.

## Conflits d’intérêts

Les auteurs n’ont aucun conflit d’intérêts à déclarer.
